# Hallucinations in Parkinson’s disease: new insights into mechanisms and treatments

**DOI:** 10.47795/ONNS5189

**Published:** 2020-07-13

**Authors:** Rimona S Weil, Suzanne Reeves

## Abstract

Hallucinations are common in Parkinson’s disease and can be distressing to patients and their families. They are associated with higher rates of nursing home placement and with increased mortality. Their underlying mechanisms have been elusive, but recent advances in network imaging provides some intriguing insights into possible underlying drivers. Treatment is complicated by risk of worsening Parkinson’s motor symptoms and by higher rates of mortality with antipsychotics, but new therapeutic avenues are emerging that offer potential hope.

Visual hallucinations are common in Parkinson’s disease, affecting up to 75% of patients over the disease course. The emergence of visual hallucinations has a significant impact on the quality of life of both patients and their families: they are strongly associated with cognitive decline and increased mortality and they are the strongest predictor of earlier placement in care homes.^1^

Although often initially benign and even entertaining, they can become distressing with disease progression, when insight is lost, and when associated with depression or delusions. They almost invariably involve perception of people and animals, often in vivid detail, with patients describing scenes of Victorian women and small children playing. They often occur at specific times of day, usually in the evening, and in specific places, usually in the patient’s own home. At early stages of disease, patients can describe minor hallucinations with misinterpretation of innocuous objects such as piles of clothes as dogs and cats. They also experience passage hallucinations, which involve the illusion of objects passing across the peripheries of vision^2^ and extracampine hallucinations, or the sense of a presence.

Less frequently, patients have hallucinations in other modalities as the illness progresses, although these are usually less well-formed. For example, auditory hallucinations in people with Parkinson’s disease are largely non-verbal, with muffled, undistinguishable sounds. Occasionally patients describe tactile, gustatory or olfactory hallucinations, which tend to co-occur with visual hallucinations.

Visual hallucinations pose a particular challenge in Parkinson’s disease as the very treatments for motor symptoms in Parkinson’s disease can also trigger and worsen hallucinations. Finding treatment for hallucinations that are both effective and safe is an area of great unmet need as antipsychotic drugs worsen Parkinson’s symptoms, are associated with cognitive deterioration and an increased risk of stroke and death.^3^

## Mechanisms

### Network changes and thalamic drivers

Visual hallucinations have fascinated neurologists and neuroscientists for many years, with their tantalisingly rich and often narrative detail. Due to their transient nature, they have been challenging to investigate, with no clear mechanism found, but many theories have been proposed. Previous models for visual hallucinations considered them as “cortical release” phenomena, where spontaneous activity occurs in the absence of visual stimuli. Alternative models suggested that hallucinations arise due to incorrect binding of objects into visual scenes.^4^

Advances in computational modelling and network neuroscience have opened up approaches to understanding the brain in new ways. Recent models suggest that Parkinson’s hallucinations could arise due to a shift in dominance of difference networks. Specifically, there is thought to be a breakdown in those networks directed to attention and perception, and overactivity of the default mode network (DMN),^5,6^ a large-scale network that becomes activated during rest, and in day dreaming and mind-wandering. Indeed abnormal levels of default mode network activation are seen in patients with Parkinson’s hallucinations.^7^

**Figure 1:**
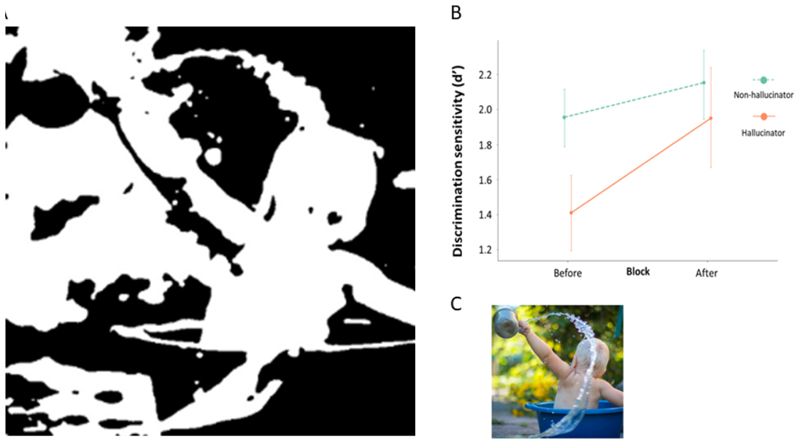
Patients with visual hallucinations over rely on prior information. A. Two-tone test image shown to patients with visual hallucinations. B. Greater improvement in image recognition is seen in Parkinson’s hallucinators than those with no hallucinations after viewing the template image. C. Template colour image, from which the two-tone image was generated. Adapted from Zarkali A, Adams RA, Psarras S, Leyland LA, Rees G, Weil RS. *Increased weighting on prior knowledge in Lewy body-associated visual hallucinations.* Brain Commun. 2019;1(1):fcz007. doi:10.1093/braincomms/fcz007

Related to this is the theory that hallucinations arise as a result of failure to integrate sensory information with prior knowledge^8^ and we recently showed that patients with Parkinson’s who hallucinate over rely on prior knowledge compared with those that do not hallucinate (See Figure 1).^9^ In this way, hallucinations arise due to over interpretation of visual input. The thalamus is likely to be important as a driver of shifting network control, and release of DMN inhibition.^6^ Consistent with this, we recently showed reduced white matter connectivity in posterior thalamic projections in patients with Parkinson’s hallucinations.^10^

### Neurotransmitters and hallucinations

The role of dopamine in the pathophysiology of psychotic symptoms has long been studied. In Parkinson’s disease, it is recognised that visual hallucinations increase with the dose and duration of levodopa treatment,^11^ and that dopamine agonists are linked with higher rates of visual hallucinations.^12^ It has been suggested that hypersensitisation of nigrostriatal dopaminergic neurones by anti-Parkinson’s drugs is an important extrinsic contributor to visual hallucinations.^13^

Visual processing involves a complex interplay between dopaminergic, serotonergic, cholinergic, and GABAergic neurons^14^ and disruption of this dynamic balance, due to intrinsic, disease-related changes underpins the emergence of visual hallucinations in Parkinson’s disease. The distinct contribution of each neurotransmitter has however proved difficult to disentangle, due to the overlapping functional networks involved in the interpretation of visual stimuli.^14^

Perceptual inference (filling in the gaps in what our senses tell us) relies on the brain’s ability to make accurate predictions about the reliability of sensory data. The thalamoreticular nucleus, a shell of GABA-releasing neurones surrounding the thalamic circuits, plays a key role in perceptual inference, as it modulates information flow to facilitate salient stimuli and suppress less relevant stimuli.^15^ Acetyl choline is a critical modulator of the thalamoreticular nucleus via nicotinic alpha 7 and muscarinic M2 receptors, and acts as a ‘sensory precision signal’.^16^

The serotonergic system is involved in early sensory processing, complex visual processing and (with noradrenaline), modulates behavioural responses (inhibition/ arousal) to visual inputs.^14^ This multiplicity of function reflects the widely distributed network and functional diversity of receptors which modulate the activity of sensory cortices, the thalamoreticular nucleus and thalamocortical circuitry. Receptor subtypes that are most closely involved in visual processing include 5HT1A (expressed on cortical pyramidal neurons), 5HT1B and 5HT2A (densely expressed in the primary visual cortex), and 5HT3 receptors (expressed on GABAergic neurones) which modulate the release of acetyl choline, dopamine and glutamate.^14,17^

Viewed in the context of ‘network control’, the early disruption of serotonergic and cholinergic neurotransmission that occurs in Parkinson’s disease may play a key role in thalamic driven decoupling of the DMN^17^ and this is a major area of research interest.

### Treatment approaches

The general principle for treating visual hallucinations in Parkinson’s disease is to look for recent triggers, such as infection, or recent medication changes. The next step is to reduce or stop medications that could be worsening hallucinations and only then to consider specific treatments.^18^ Importantly, specific interventions for visual hallucinations should only be initiated if patients are bothered by the experiences, as in most cases, side effects can outweigh benefits of treatment.

There is no evidence base for the order of withdrawal, and best practise is to withdraw whatever triggered the hallucinations. With no clear trigger, withdrawal should start with the least efficacious. A useful order has been recently provided: anticholinergics, followed by amantadine, rasagaline, dopamine agonists, monoamine oxidase (MAO) B inhibitors, entacapone and then levodopa.^19,20^

Cholinesterase inhibitors are widely thought to reduce hallucinations. However, there are no supportive randomised controlled trial (RCT) data where visual hallucinations have been the treatment indication or primary outcome. The best evidence for their benefit is the observation that Rivastigmine improved cognitive performance most in those patients with visual hallucinations.^21^

Treatment options for persistent hallucinations are limited to antipsychotic drugs, which are associated with significant side-effects (falls, sedation, worsening of cognitive and motor function) and increased mortality. National Institute of Clinical Excellence (NICE 2017, www.nice.org.uk/) guidance supports use of quetiapine, as it is safer than other atypical antipsychotics (odds ratio of mortality 2.16 compared to 2.79 for olanzapine).^3^ However the evidence for its use is weak as RCTs have shown no superiority over placebo, apart from one study which excluded patients with delusions.^22^

Clozapine has the strongest evidence for efficacy in treating distressing hallucinations in Parkinson’s disease. Two good size RCTs have shown effectiveness in reducing hallucinations and associated delusions, with no worsening of Parkinson’s motor symptoms, following very low dose treatment (10% of the dose used to treat schizophrenia).^23,24^ However, concern of agranulocytosis, daily pulse and blood pressure checks, and weekly blood monitoring for the first 18 weeks, make this impractical outside of specialist mental health settings.

Serotonergic agents have attracted considerable attention as candidate drug treatments for hallucinations, as they offer an alternative approach that is not mediated via direct antagonism of dopamine D2/3 receptors. The 5HT2A inverse agonist, Pimavanserin, was recently shown in a large randomised controlled trial to improve psychosis and visual hallucinations in Parkinson’s disease,^25^ with greatest improvement observed in patients with poorer cognition.^26^ Initial concerns of higher rates of mortality were shown to be no higher than those in this already frail patient group.^27^

An alternative serotonergic agent is the 5HT3 antagonist, ondansetron, which is already in use and licensed as an anti-emetic. Ondansetron showed early promise in the early 1990s as treatment of severe, persistent visual hallucinations in a case series of people with Parkinson’s disease^28^ and a subsequent open study, with marked improvement in hallucinations and delusions.^29^ At the time, the then high cost of ondansetron prevented further studies, but this is no longer the case and the first placebo-controlled trial of ondansetron as a Parkinson’s hallucinations treatment (TOP HAT) is planned for Autumn 2020, funded by Parkinson’s UK.

## Supplementary Material

Rimona Weil

Suzanne Reeves

Book Reviews
