# Supplementary figures and images for: Hallucinations in Parkinson’s disease: new insights into mechanisms and treatments
Source: Adv Clin Neurosci Rehabil. Author manuscript; Available in PMC 2020 Oct 23. (PMC7116251; doi:10.47795/ONNS5189)

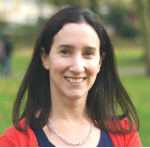

Supplement: Rimona Weil [file EMS98466-supplement-Rimona_Weil.png]

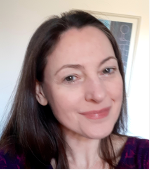

Supplement: Suzanne Reeves [file EMS98466-supplement-Suzanne_Reeves.png]
