# Supplementary material for: Hallucinations in Parkinson’s disease: new insights into mechanisms and treatments
Source: Adv Clin Neurosci Rehabil. Author manuscript; Available in PMC 2020 Oct 23. (PMC7116251; doi:10.47795/ONNS5189)
Supplement: Book Reviews [file EMS98466-supplement-Book_Reviews.pdf]

Oxford Handbook of Rehabilitation Medicine. 3rd Edition

In the best tradition of Oxford Handbooks, this is a small book that packs a heavy punch. This third edition represents a significant expansion in scope and detail compared to the second edition of 2009. With over 650 pages miraculously compressed into less than two and a half centimetres, this edition adeptly fulfils its role in providing for jobbing clinicians a succinct, easily navigable overview of key clinical topics at their fingertips.

The volume is structured in two sections, the first of which 'Common Clinical Approaches' provides 25 chapters on cross-cutting areas from Communication, to Chronic Pain, Sexual Function and Mobility and Gait. Section 2 addresses 'Condition-Specific Approaches' in 16 chapters including Traumatic Brain Injury, Multiple Sclerosis, Prolonged Disorders of Consciousness and Amputee Rehabilitation. Where there is overlap, this is usefully signposted within the text, directing the reader to other relevant chapters. The text is helpfully presented, easy to scan and interspersed with many useful illustrations and diagrams.

The devotion of two chapters to musculoskeletal conditions, as well as the inclusion of Cancer Rehabilitation and Geriatric Rehabilitation reflects the editors' timely desire to see the focus of rehabilitation as a medical specialty in the UK broaden to address conditions of greatest population burden. They will also be useful to rehabilitation practitioners in the traditional areas of neurological and spinal cord rehabilitation who find themselves working with increasingly complex conditions and co-morbidities.

Between the second and third editions the title has shifted from Clinical Rehabilitation to Rehabilitation Medicine, which is indicative of a change of emphasis towards greater rigorous pathophysiological detail and is accompanied by an authoritative, brisk editorial style. Medics, from students and junior doctors to specialists in Rehabilitation Medicine (but also Geriatrics, Neurology, Stroke Medicine and beyond) will be the main users of this handbook, but it also has much to offer to the whole multi-professional rehabilitation team.

The chapter authors are largely drawn from the UK and Australia, and sections on models of care and organisation of services reflect those settings. Inevitably, some details have already been superseded by new guidelines, for example that in TIA risk stratification, but in general this edition does an excellent job of succinctly bringing the reader up to date, and signposting further reading.

In its main aim of concisely presenting both the core principles and practical clinical details of Rehabilitation Medicine practice across an expanded scope of conditions, this handbook has certainly succeeded and it will undoubtedly become a familiar sight in MDT rooms and doctors' offices across the rehabilitation landscape.

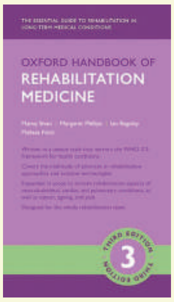

Editors: Manoj Sivan, Margaret Phillips, Ian Baguley, Melissa Nott
Published by: Oxford University Press
Paperback price: £34.99
Pages: 658
ISBN: 9780198785477
Reviewed by:
Stephen Halpin,
Senior Clinical Research Fellow and Consultant in Rehabilitation Medicine,
Academic Department of Rehabilitation Medicine,
University of Leeds.
